# Blackcurrant (*Ribes nigrum* L.) and Kamchatka Honeysuckle (*Lonicera caerulea* var. Kamtschatica) Extract Effects on Technological Properties, Sensory Quality, and Lipid Oxidation of Raw-Cooked Meat Product (Frankfurters)

**DOI:** 10.3390/foods10122957

**Published:** 2021-12-01

**Authors:** Lukáš Jurčaga, Marek Bobko, Adriana Kolesárová, Alica Bobková, Alžbeta Demianová, Peter Haščík, Ľubomír Belej, Andrea Mendelová, Ondřej Bučko, Miroslav Kročko, Matej Čech

**Affiliations:** 1 Institute of Foods Sciences, Faculty of Biotechnology and Food Sciences, Slovak University of Agriculture in Nitra, Trieda Andreja Hlinku 2, 94976 Nitra, Slovakia; xjurcaga@uniag.sk (L.J.); alica.bobkova@uniag.sk (A.B.); xdemianova@uniag.sk (A.D.); peter.hascik@uniag.sk (P.H.); lubomir.belej@uniag.sk (Ľ.B.); andrea.mendelova@uniag.sk (A.M.); miroslav.krocko@uniag.sk (M.K.); xcech@uniag.sk (M.Č.); 2 Institute of Applied Biology, Faculty of Biotechnology and Food Sciences, Slovak University of Agriculture in Nitra, Trieda Andreja Hlinku 2, 94976 Nitra, Slovakia; adriana.kolesarova@uniag.sk; 3 Institute of Animal Husbandry, Faculty of Agrobiology and Food Resources, Slovak University of Agriculture in Nitra, Trieda Andreja Hlinku 2, 94976 Nitra, Slovakia; ondrej.bucko@uniag.sk

**Keywords:** blackcurrant, honeysuckle, meat product, lipid oxidation, sensory quality, frankfurter sausage

## Abstract

Oxidation is one of the most prevalent factors responsible for meat product deterioration. Due to their potential health risks, commonly used synthetic antioxidants are beginning to be frowned upon by customers. The industry is searching for a natural replacement. In our study, we incorporated blackcurrant (*Ribes nigrum* L.) and Kamchatka honeysuckle (*Lonicera caerulea* var. Kamtschatica) extracts into raw-cooked meat products (frankfurters) as natural antioxidants. We observed that both extracts at concentrations of 3 mL·kg^−1^ were able to significantly (α = 0.05) postpone lipid oxidation in our samples, with results comparable to vitamin C (0.5 mg·kg^−1^) addition. Moreover, we did not observe negative effects of the extracts on the product’s color, pH, or textural properties. Negative results were reported in the sensory evaluation of honeysuckle addition samples. This could have been caused by the natural strong and bitter taste of honeysuckle, which was transferred to the extracts and, subsequently, into the meat product.

## 1. Introduction

Meat products are food products prepared from meat and other edible parts of animals that are used for slaughter, ingredients, and various delicacies. They represent processed meat and other raw materials that feature specific shape and organoleptic properties. They dominate in the proportion of meat raw material, which is supplemented with fats and non-meat components. They are intended for direct consumption or further heat treatment before consumption [1]. The oxidation of the lipid component of food is a severe problem in the food industry, as it leads to shortened shelf life and a deterioration in the taste and functional and nutritional properties of food products, as well as possible health risks [2]. The loss of organoleptic properties is caused by the formation of undesirable substances, which is itself caused by the degradation of unsaturated fatty acids during autooxidation. Fats and oils are contained in almost all agri-food raw materials and are a dominant component in many food products. All foods, even those with low lipid content, are very susceptible to oxidative damage by active forms of atmospheric oxygen, especially when exposed to light [3]. In meat and meat products, autooxidation is a fundamental mechanism of lipid oxidation. Autooxidation is the oxidative degradation of unsaturated fatty acids by an autocatalytic process based on the mechanism of radical chain reactions. Chain radical oxidation generally occurs as a three-phase process involving the initiation, propagation, and termination phases. In particular, reactions in the propagation phase are responsible for autooxidation’s autocatalytic nature [4].

Comminuted (ground) cooked meat products (gel/emulsion systems) are a commercially important group of meat products, of which frankfurters are among the more popular varieties. Frankfurters are frequently consumed meat products, which enjoy wide consumer acceptance in certain sections of the global population [5]. Frankfurters can contain up to 30% fat, with an industry average of about 20%, and salt concentrations of around 2% and higher [6]. Consumers demand low-fat products, mainly meat products with lower saturated fatty acids, which are also recommended by institutions such as the Food and Drug Administration (FDA) and the World Health Organization (WHO), as well as researchers [7]. There are many strategies to improve frankfurters’ fatty acid profiles (decreasing the SFA and increasing the MUFA and PUFA levels that make products more susceptible to oxidation). Many of them are oriented around the addition of various vegetable substances in place of meat fats [8,9,10]. Synthetic and natural antioxidants are used for the reduction of the oxidation process in meat. There is increasing demand for natural antioxidants due to the safety and toxicity issues with synthetic antioxidants [11]. The growing interest in natural foods has forced the food industry to introduce natural antioxidants in food products to delay the oxidative degradation of lipids, improve foods’ quality and nutritional value, and replace synthetic antioxidants [12]. Natural antioxidants can protect biologically critical cellular components from oxidative processes caused by reactive oxygen species [13].

Blackcurrant *(Ribes nigrum* L.) is a woody deciduous shrub with dark-colored berries, belonging to the family *Grossulariaceae*. Blackcurrants come from northern and central Europe and Asia and are grown for ornamental purposes and berry production in Europe and North America [14]. Compared to other berries, such as gooseberries, strawberries, red currants, raspberries, and white currants, blackcurrant fruits feature higher concentrations of polyphenols, especially phenolic acid derivatives, anthocyanins, flavonols, condensed tannins (proanthocyanidins), flavan-3-ol (catechins), and hydrolyzable tannins (ellagitanines and gallothanines). Thus, blackcurrants are considered a cost-effective and profitable fruit to be used as an ingredient in a healthy diet [15]. The *Lonicera* species consists of about 200 species, including ornamental, medicinal, and food plants. One of the most economically important species is *Lonicera caerulea* L. (blue honeysuckle), which bears blue-violet-to-dark blue fruits. This species is widespread in the northern hemisphere, and is particularly abundant in continental climate zones. It primarily originates in Russia, Canada, China, and Japan. The quality profile of anthocyanins is specific and characteristic for earthworm fruits, consisting in the predominant compound cyanidine-3-glucoside (up to 90%), followed by cyanidine-3,5-diglucoside, peonidine-3-glucoside, and other minor components—anthocyanins. Quantitative profiles vary significantly depending on horticultural and climatic conditions, or growing area [16]. The same authors have determined that the vegetation year also affects the total amount of secondary metabolites. A variety of species growing in different climatic zones feature different phytochemical compositions within the same genotype [17].

Various studies focus on the addition of natural extracts into meat products to replace synthetic antioxidants. Their authors used a wide range of extract sources, such as red grape pomace, pomegranate rind powder, banana peel, peanut skin, green tea, and many others. All the authors reported a positive effect of extracts on TBARS values in their respective meat products during storage [18,19,20,21,22]. Jia et al. [23] used blackcurrant extract and reported the strong antioxidant effect of its addition on the quality of pork patties.

This research aimed to explore the antioxidant ability of two plant extracts in meat products (frankfurters). Both blackcurrant and honeysuckle are well known as rich sources of polyphenols with strong antioxidant potential. We tested the antioxidant ability when incorporated into a raw-cooked meat product to evaluate the effect of natural antioxidants on lipid oxidation, sensory quality, and microbiological stability.

## 2. Materials and Methods

The whole experiment was carried out twice to confirm or to refute the obtained results. In every experiment run, every measurement was performed for five repetitions. All the results are the average values from both experiments. The plant material of species blackcurrant (*Ribes nigrum* L.) and honeysuckle (*Lonicera caerulea* var. kamtschatica) was provided by Botanic Garden of Slovak University of Agriculture in Nitra. The meat (loin and shoulder) for the meat product manufacturing were bought at a local butcher shop.

### 2.1. Extract Preparation

The extraction of both blackcurrant and honeysuckle was performed according to Shirahigue et al. [24]. Dried and homogenized fruits (20 g) with 100 mL of 80% ethanol were mixed together in a shaker and allowed to rest for 24 h, in the dark, at room temperature. The filtrate was filled up to 100 mL with ethanol. The liquid fraction was then evaporated until dry at 65 °C in a vacuum rotary evaporator. The weighed dry residue was redissolved in 50 mL of water. The final extract was stored at 4 °C, in the dark.

### 2.2. Moisture

The moisture content of the dried berry samples was determined using the KERN DAB 100-3 moisture analyzer (KERN & SOHN GmbH, Balingen, Germany) and expressed as a percentage of moisture. The drying program was set at 110 °C.

### 2.3. Determination of Total Antioxidant Capacity (TAC)

The antioxidation activity measurement was performed by the DPPH (2,2-diphenyl-1-picrylhydrazyl) radical method described by Brand-Williams et al. [25]. Exactly 0.025 g of DPPH was weighed and then dissolved with ethanol and the volumetric flask with the stock solution was filled up to 100 mL. The stock solution was diluted with methanol (1:9). The diluted 3.9 mL DPPH solution was poured in glass cuvettes and the initial DPPH absorbance (A_0_) was measured at a wavelength of 515.6 nm. Next, 100 μL of sample extract was pipetted into a cuvette and the mixture was stirred with a glass stick. The absorbance (A_t_) was measured after 10 min at 515.6 nm (T80 UV/VIS Spectrometer; PG Instruments, Ltd.; Lutterworth, UK). We determined the antioxidant activity of the extracts as a percentage of the inhibition of DPPH radicals. The scavenging capacity was calculated using the following equation:

$$\%\;\mathrm{inhibition}\;\mathrm{of}\;\mathrm{DPPH}=\frac{{(A}_{0}-A_{s})-{\;(A}_{t}-A_{s})}{{(A}_{0}-A_{s})}\;\text{×}\;100$$

where A_0_ is the initial absorbance of DPPH solution,

A_s_ is the absorbance of ethanol (blank), and

A_t_ is the absorbance after 10 min.

### 2.4. Determination of Total Polyphenols Content (TPC)

The extracts were prepared and tested TPC. For this purpose, we used the Folin–Ciocalteu reagent method, in accordance with Fu et al. [26]. The content was expressed as grams of Gallic acid equivalents (GAE) per kilogram of dry fruit weight. For the measurement, a double-beam UV-VIS spectrophotometer (T80 UV/VIS Spectrometer; PG Instruments, Ltd.; Lutterworth, UK) equipped with an 8 place cuvette holder was used. The glass cuvettes were type S/G/10 (Exacta+Optech GmbH, Munich, Germany).

For the standard preparation, exactly 100 mg gallic acid were weighed (Analytical balance Sartorius TE214S-0CE, Sartorius Lab Instruments GmbH & Co. KG, Göttingen, Germany) and diluted with demineralized water up to 100 mL volume to prepare a stock solution. One mL of the stock solution was diluted with distilled water up to 200 mL volume. The calibration curve was prepared in range of 5–200 mg·L^−1^ of gallic acid. The blank contained Folin–Ciocalteu reagent and distilled water, without the standard or extract. The correlation coefficient of the calibration curve reached R^2^ = 0.996.

For the samples’ preparation, 50 μL were pipetted into 50 mL volumetric flasks; subsequently, 2.5 mL of Folin-Ciocalteu reagent diluted with distilled water (1:2 *v/v*) were added. Next, 5 mL of Na_2_CO_3_ (20% water solution) was added. Following this, flasks were filled with distilled water. In this form, the samples were left for 2 h at a room temperature to develop the blue complex. The measurement wavelength was set to 765 nm.

### 2.5. Determination of Total Anthocyanins (TA)

The measurement of the total anthocyanins content was carried out in accordance with Lapornik et al. [27] One milliliter of extract was pipetted into two tubes. One mL of 0.01% HCl solution in 95% ethanol was added into each tube. Next, (A1) 10 mL of 2% aqueous HCl solution were added into the first tube, and into another tube we added (A2) 10 mL of solution with pH = 3.5 (prepared from 0.2 M Na_2_HPO_4_ and 0.1 M citric acid). The absorbances of both samples were measured at 520 nm against a blank sample (water). The total anthocyanin content was calculated as follows, where “f” is constant (396.598). For the measurement, a double-beam UV-VIS spectrophotometer (T80 UV/VIS Spectrometer; PG Instruments, Ltd.; Lutterworth, UK) was used.

$$\mathrm{Content}\;\mathrm{of}\;\mathrm{Total}\;\mathrm{Anthocyanins}\;\left(\mathrm{mg}.L^{-1}\right)\;=\;\left(A1-A2\right)\times \;f$$

### 2.6. Frankfurter Preparation

For the preparation of the meat product, we used the following ingredients: pork meat, water, salting mixture with 0.3% sodium nitrite concentration, black pepper, sweet and spicy paprika, and nutmeg. The composition of the frankfurters is listed in Table 1. All the ingredients were mixed together, and the antioxidants were added. The control group (Con-0) was prepared with no antioxidant additive at all. The second group (Con-C) contained 0.5 g of citric acid. The third (BCE-1) and the fourth group (BCE-2) contained 3 mL and 5 mL extracts of blackcurrant, respectively. The fifth (KHE-1) and the sixth (KHE-2) were accorded 3 mL and 5 mL, respectively, of Kamchatka honeysuckle extract. Th finished pork frankfurters were heat-cured by wet smoking to obtain a temperature of 70 °C in the core for at least 10 min, cooled down, and packaged, vacuum sealed, and stored at 4 °C for 21 days.

### 2.7. pH Measurement

The measurement of the pH was carried by using a benchtop pH meter (Orion Star™ A211 Benchtop pH meter, Beijing, China) with a piercing probe. We calibrated the pH electrode using a three-calibration solution (Hamilton AG Bonaduz, Bonaduz Switzherland with pH 4, 7 and 10) at a temperature of 20 °C. Before measuring the pH of the product, the frankfurters were removed from chilled storage and allowed to warm up to 20 °C, since this temperature was used during the calibration. The measurement of the pH was carried out on the 1st, 7th, 14th, and 21st day.

### 2.8. Color Determination

Every sample was homogenized, and color measurement was conducted by spectrophotometer (Konica Minolta CM-2600d, Osaka, Japan) with the setting Specular Component Included (SCI). We used the D65 light source and a 10° observer, with a port 8 mm in diameter. The white plate calibration was performed at 23 °C, as suggested by the manual. The results were coordinates in the color interface of CIELab, where L* represents lightness, a* represents redness-greenness, and b* represents yellowness-blueness. From the obtained results, the chroma was calculated by the following equation: chroma = square root of (a*² + b*²). The color measurement was carried out on the 1st and the 21st day.

### 2.9. Texture Analysis

To determine the textural properties, a texture analyzer machine (TA.XTplus Texture Analyser, Godalming, UK) was used. The frankfurters were heated before conducting the analysis to a core temperature of 70 °C. Before the analysis, the samples were cut into blocks with a base 1 × 1 cm. We used default options for the hot-dog analysis with a Warner-Bratzler probe (V-blade) selected from the analyzer library. The firmness and toughness parameters were observed. The texture analysis was carried out on the 1st, 7th, 14th, and 21st day.

### 2.10. Microbiological Examination

The microbiological examination was carried out by using a simple dilution method. A microbial analysis was performed during storage, on the 7th, 14th, and 21st day. We observed the following microorganisms: psychrotrophic bacteria, *Bacillus* sp., *Enterococcus* sp. and *Lactobacillus* sp. All the microorganisms were inoculated on their respective nutrient medium and incubated accordingly:

- Psychrotrophic bacteria—PCA agar, 10 days, 6.5 ± 1 °C,
- *Bacillus* sp.—M-PA agar, 5 days, 25 °C ± 1 °C,
- *Enterococcus* sp.—M-PA agar, 3 days, 37 °C ± 1 °C,
- *Lactobacillus* sp.—MRS agar, 5 days, 37 °C ± 1 °C.

To determine the colony-forming unit, the plate counting method was used and subsequently calculated, and expressed as log cfu·g^−1^.

### 2.11. Sensory Evaluation

The sensory evaluation was performed by a trained panel on the 7th, 14th, and 21st day after the preparation. All the samples were heated before they were evaluated. Five sensory parameters were observed: appearance (surface and on a cut), color, odor, consistency, and taste. Every parameter was evaluated on a 5 point scale, according to which 5 is the best and 1 is the worst score for the selected parameter. Together, the product could obtain 25 points at best. The sensory panel consisted of 10 trained evaluators, 25–50 years of age, of both genders. All the evaluators were from the Department of Technology and Quality of Animal Products with experience of evaluating animal product quality.

### 2.12. Determination of Oxidative Stability

The oxidative stability of the raw-cooked product was based on measurements of the malondialdehyde (MDA) concentration by thiobarbiturate test using a 2-thiobarbituric acid (TBA) solution. This solution was prepared using 2.1623 g of TBA, to which we added 125 mL of distilled water (in 250 mL volumetric flask). We placed the volumetric flask in a water bath and heated the solution at 90 °C until the TBA dissolved. Subsequently, the solution was cooled down, and 15 mL of 1 mol·L^−1^ NaOH and 3 mL of 1 mol·L^−1^ HCl were added, and made up to 250 mL with distilled water. For the analysis, we used 1.5 g of a sample. Exactly 1 mL of EDTA and 5 mL of 0.8% butylated hydroxytoluene solution (BHT) were added to the sample and mixed gently. Next, we added 8 mL of 5% trichloroacetic acid solution (TCA) and homogenized sample for 30 s at 10,000 rpm (IKA T 18 digital ULTRA-TURRAX^®^, Staufen, Germany). The sample was then left to chill for 10 min and centrifuged at 3500 *g* at a temperature of 4 °C for five minutes (Hettich Universal 320, Tuttlingen, Germany). The top hexane layer was separated from the sample and discarded. The rest of the sample was filtrated using a Whatman 4 grade filter (Chemlab, Barcelona, Spain) into the 10 mL volumetric bank and made up to the volume with 5% TCA solution. A total of 4 mL of the prepared sample was mixed with 1 mL of 2-thiobarbituric acid (TBA) solution, incubated in a water bath at a temperature of 70 °C for 90 min and left to cool down to room temperature for 45 min. As the next step, the absorbance of the sample was measured at a wavelength of 532 nm (T80 UV/VIS Spectrometer; PG Instruments, Ltd.; Lutterworth, UK). A calibration curve was used for the calculation of the results. These were expressed as the quantity of malondialdehyde (MDA) (mg) present in 1 kg of sample. MDA concentration measurements were carried out on the 1st, 7th, 14th, and 21st day.

### 2.13. Statistical Analysis

To compare the results of the individual analyzed groups, ANOVA analysis with Duncan test was used. For all the tests, the level of signification α was set to 0.05. To perform the analysis, XLSTAT software was used (XLSTAT Addinsoft, statistical and data analysis solution, 2021, New York, NY, USA).

## 3. Results and Discussion

### 3.1. Extracts Examination

To better understand the extracts’ effects on certain meat products, selected chemical characteristics were observed. The DPPH scavenging activity was determined on the day after the extraction. When compared, the honeysuckle extract was observed to be more effective at scavenging than the extract of blackcurrant berries. Furthermore, both extracts were subjected to the determination of their Total Anthocyanin (TA) and Polyphenol Content (TPC). As expected, Kamchatka honeysuckle contains a higher concentration of anthocyanins than blackcurrant. The blackcurrant extract also exhibited lower TPC. All the results of the extract examination are shown in Table 2.

### 3.2. Frankfurters Examination

#### 3.2.1. pH Measurement

The measurement of the pH values was carried out during the whole storage period in all the experimental groups. The control group (Con-0) and all the experimental groups exhibited similar pH values, ranging from 6.15 ± 0.08 to 6.28 ± 0.12. Furthermore, all the groups showed very minor changes over the storage time without any significant differences (α = 0.05). On the other hand, the control group with vitamin C showed significantly (α = 0.05) lower pH values than all the other groups. The initial pH of the Con-C group was only 5.48 ± 0.9. On a later day of storage, there was a growth, albeit only up to 5.67 ± 0.06. Various authors have experimented with berries extract in meat products. Kumar and Kumar [28] used *Murraya koenigii* berry extract in chicken meat batter. Unlike us, they observed differences between the control and the experimental group. Furthermore, Tamkutė et al. [29] observed a decrease in pH in pork patties after adding chokeberry extract. Furthermore, Jaberi et al. [30] tested the addition of barberry extract into chicken frankfurters. The authors reported that the use of *Berberis vulgaris* extract in frankfurters exerted a very significant effect on the pH (*p* < 0.01). Our obtained pH values are shown in Table 3.

#### 3.2.2. Color Analysis

The color determination of all the samples was carried out on the 1st and 21st day of storage. The aim was to monitor changes between the negative control (C-0 group without antioxidant) and the experimental groups and changes over time. We observed a statistical difference between the negative control and all the groups with antioxidants in the lightness parameter on the first day, where the negative control displayed the highest value. There was no redness difference, and only the KHE-2 group showed lower yellowness values than the negative control. At the end of the storage period, on the 21st day, the lightest sample of all was the control with vitamin C, which demonstrated the best ability to postpone the darkening process of the frankfurter sausages. Significant differences were observed in the redness parameter; however, only Con-C group was statistically different. All the experimental groups reached almost identical values. The highest yellowness score was achieved by the Con-C group and the lowest by the KHE-2 group. Various authors have observed extract color changes in meat products with natural extract addition. Selani et al. [31] reported that the addition of grape seed extracts caused darkening and lower intensities of red and yellow in the meat products. The sensory evaluation revealed no significant alteration in the odor and flavor scores of the samples treated with extracts. Jia et al. [23] reported a steady decrease in lightness and redness in pork patties with blackcurrant extract during 9 days of storage. However, the authors did not use vacuum packaging for their product. Xia et al. [32] observed a decrease in redness and increased yellowness, correlated with increased lipid oxidation. These results suggest that the yellowness in pork may be due to non-enzymatic browning reactions between lipid oxidation products and the amine in meat. However, we did not observe any decrease in redness regarding lowered MDA concentrations in the experimental groups. In these findings, we are in line with the results obtained by Nowak et al. [33], who reported that the yellowness of the sausages in their experiment was most likely due to the color of the plant extracts from blackcurrant and sour cherry. Regarding the color changes of heat-treated products, Chung et al. [34] used blackcurrant in the treatment of the Korean beef specialty, Hanwoo Tteokgalbi. The authors reported that the treated samples were brighter and redder than the untreated control group. The treated samples were also reported to be preferred by consumers in a sensory evaluation. The color measurement results we obtained are listed in Table 4.

#### 3.2.3. Textural Analysis

**Figure 1:**
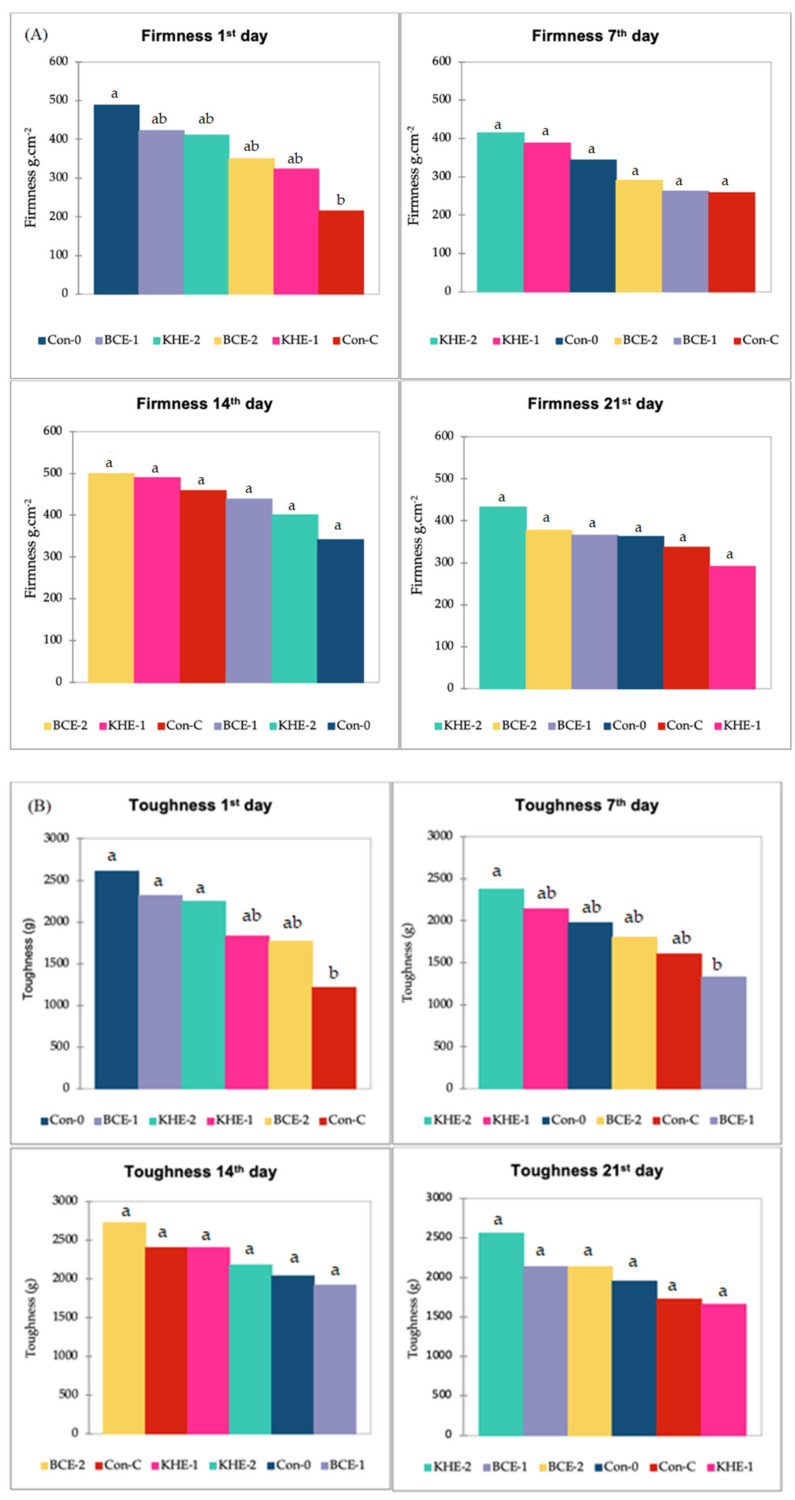
(**A**) Results of textural analysis (firmness) ANOVA comparation by Duncan test; (**B**) Results of textural analysis (toughness) ANOVA comparation by Duncan test. Note: a,ab,b represents statistically significant differences (α = 0.05) between samples on every day of measurement in both firmness (g) and toughness (g·s); Con-0 = negative control, Con-C = control with 0.5 g vit. C, BCE-1 = 3 mL blackcurrant ext. addition, BCE-2 = 5 mL blackcurrant ext. addition, KHE-1 = 3 mL honeysuckle ext. addition, KHE-2 = 5 mL honeysuckle ext. addition.

The textural analysis was carried out on the 1st, 7th, 14th, and 21st days of storage. All the samples were cut into blocks with a base of 1 × 1 cm. Our aim was to determine whether the extract addition would affect the firmness and toughness of the final meat product over storage time. Firmness is a descriptive term that has no standard scientific definition. However, in textural analysis it is accepted as being either the force at the maximum distance of a compression cycle or, as in our case, as the maximum force reached prior to a fracture. Our analysis did not observe any significant (α = 0.05) difference in firmness among all the groups throughout the storage period (Figure 1A). Toughness is described as a force in time, which is needed to cut through the whole piece of a sample. In this parameter, certain differences were observed mainly between the negative control and the samples with antioxidant addition. These differences were statically significant on the 1st and 7th day (Figure 1B). This could be a sign that antioxidant addition helps to preserve the tenderness of sausages in the early storage period. Differences among the analyzed groups are more likely to have been caused by the technological process of batter filling than by the addition of antioxidants.

#### 3.2.4. Microbial Examination

Another essential aspect is the microbiological safety of meat products. During our analysis, we performed a microbiological examination of the samples three times during the storage, on the 7th, 14th, and 21st day. We did not observe any *Enterococcus* sp. occurrence in the analyzed groups during the whole 21 days. *Enterococcus* sp. is mainly a sign of secondary contamination, which we were able to avoid. The other observed groups (psychrotrophic bacteria, *Bacillus* sp., *Lactobacillus* sp.) demonstrated a time-dependent increase during the storage period (Table 5). Among all the experimental groups, we did not observe any significant difference (α = 0.05) in any of the three microorganism species. We did not observe any anti-microbial effect of blackcurrant or honeysuckle extract in the concentrations applied in our study. Similarly, low antimicrobial abilities of blackcurrant were reported by Puupponen-Pimiä et al. [35]. The authors claimed that out of all their used fruit extracts, blackcurrant was the least effective against gram-negative bacteria. For example, *Escherichia coli* strain 50 was sensitive to all phenolic extracts except blackcurrant. Furthermore, blackcurrant slightly stimulated the growth of *Lactobacillus rhamnosus* VTT E-97800 and GG VTT E-96666, and *Lactobacillus paracasei*. Furthermore, Nohynek et al. [36] reported that phenolic extracts of black currant, lingonberry, cranberry, and buckthorn berry are not active against *Salmonella*, whereas lyophilized whole berries are effective and feature growth-inhibiting activity. Our obtained data demonstrate that either of the used extracts offer an antimicrobial effect when incorporated into the raw-cooked meat product. The results of our microbial examination are listed in Table 5.

#### 3.2.5. Sensory Evaluation

The sensory quality of the product is possibly the most crucial parameter for customer satisfaction. Any experimental additive must not alter quality markers, such as taste or odor. In our experiment, a sensory evaluation of the frankfurter sausages was carried out on the 7th, 14th, and 21st day of storage. We aimed to observe changes in the selected parameters over the storage time. After seven days, we did not observe any statistically significant difference among the groups (α = 0.05). However, the group with vitamin C addition (Con-C) showed a lower appearance score, 3.5 ± 0.7 points on average. This could have been due to vitamin C’s ability to bond water and possibly cause bubbles in the meat batter of the final product.

On the other hand, the Con-C group obtained the highest average score in color and odor parameters. The Con-0 group achieved the overall best score. From the experimental groups, the overall best score was achieved by the BCE-1 group. After fourteen days, we observed a decrease in the control Con-0 group’s score in all the parameters. The best sample for all the parameters and overall acceptability was experimental group BCE-1. This suggests that blackcurrant extract can even improve the sensory quality of meat products. The worst-performing group was the experimental group KHE-2. All the evaluators reported a more pungent, bitter aftertaste than in the first evaluation carried out after seven days. At the end of the storage period, the control group BCE-1 was again evaluated as the overall best. However, the best score in color and odor was achieved by the Con-C group. Therefore, the evaluators suggest that vitamin C features the best ability to preserve the color and aroma of frankfurter sausages.

**Figure 2:**
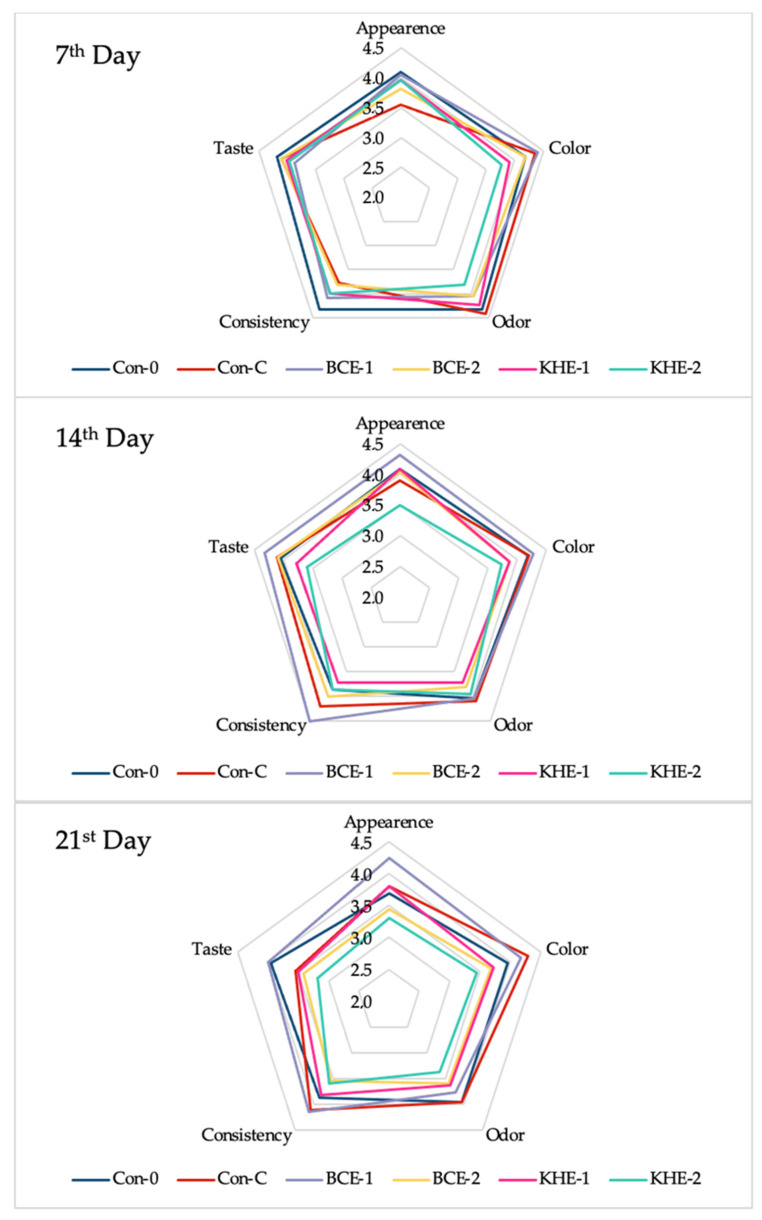
Effect of extract addition on sensory properties of analyzed frankfurter sausages.

Probably the most crucial marker is taste. Regarding this marker, the BCE-1 group received the highest score of all the groups. The experimental groups with the honeysuckle extract were again penalized because of its strong, bitter taste and aftertaste. Moreover, the higher concentration (KHE-2) of honeysuckle extract negatively affected the meat product’s color, appearance, and consistency (Figure 2). Jaberi et al. [31] incorporated barberry extract into chicken frankfurters in concentrations of 0.75, 1.5, and 3%. They reported statistical differences between the experimental groups regarding the flavor parameter (α = 0.05). Hardness, juiciness, saltiness, and general acceptability were not affected by the use of carmine in the control group or the barberry extract group (α = 0.05). Furthermore, in their evaluation, the highest color value was achieved by the group treated with 3% barberry extract. Similar results were obtained in our experiment with blackcurrant extract. These results show that extracts from berries can improve the color quality of meat products. Stobnicka and Gniewosz [37] used swamp cranberry (*Vaccinium oxycoccos* L.) fruit and pomace extract in pork burgers, which were evaluated by a sensory panel. Their obtained results indicate that adding both extracts at a concentration of 2.5% to pork burgers did not affect the sensory quality, as perceived by consumers, on a statistically significant level. Overall, natural extracts offer the potential even to enhance the sensory quality of some meat products. However, in our case, the strong natural bitter taste of honeysuckle extract altered all the markers of the experimental sausages. The obtained results are presented in Figure 2.

#### 3.2.6. Oxidative Stability

Malondialdehyde, or MDA (1,3-propanedial), is one of the most important aldehydes produced during the secondary lipid oxidation of polyunsaturated fatty acids. This aldehyde is also of great importance in meat, since it produces rancid aromas even in low amounts of meat, and it is considered the major marker of lipid oxidation [38]. In our experiment, we observed MDA growth in all the experimental groups during the storage period. From the first day, the Con-0 group showed slightly higher MDA levels than all the other groups with the addition of antioxidants. Compared with the other experimental groups, we observed a higher growth of MDA in the negative control (Con-0) after the first week. However, the differences were not statistically significant (α = 0.05). A measurement carried out on the fourteenth day revealed that both blackcurrant and honeysuckle extract and vitamin C significantly reduced the amount of malondialdehyde compared to the group without antioxidants. Among the experimental groups, no significant differences were observed. At the end of the storage period, the difference between the negative control and all the other groups markedly increased. All the experimental groups significantly (α = 0.05) retarded lipid oxidation in the analyzed samples. Differences among the experimental groups with antioxidants were also observed. Honeysuckle at a concentration of 5 mL·kg^−1^ was the most effective option for reducing the amount of MDA. The experimental groups also demonstrated that the ability to postpone lipid degradation is concentration-dependent. Measured MDA concetration are listed in Table 6. The overall percentage increase in MDA concentration among the groups during storage was as follows: Con-0 (146.8%) > BCE-1 (56.6%) > Con-C (56.5%) > KHE-1 (54.5%) > BCE-2 (41.0%) > KHE-2 (31.5%). The obtained results from the MDA measurement are in Table 3. Our findings are in line with those of Jia et al. [24]. These authors reported that the addition of blackcurrant extract in concentrations of 5, 10, or 20 g·kg^−1^ decreased the production of malondialdehyde by 74.9%, 90.6%, and 91.7%, respectively, in pork patties, compared to concentrations measured in the negative control. The authors also claimed that the efficacy of BCE was comparable with that of BHA. BCE treatments at concentration levels of 10 and 20 mg·kg^−1^ reduced MDA values similarly to 0.2 g·kg^−1^ of BHA. The results obtained by these authors suggest that the addition of blackcurrant extracts inhibits lipid oxidation by blocking radical chain reactions. Regarding protein oxidation, compared to the control group, the results showed lower protein oxidation in the analyzed patties (after 6 and 9 days of storage, in concentrations of 10 and 20 g·kg^−1^ of blackcurrant extract). Nevertheless, the positive effect was less pronounced compared to the effect on lipid oxidation. Other authors have researched various other berry extracts, with similar results. Armenteros et al. [39] evaluated lipid oxidation by TBARs values and the lipid-derived volatile compounds of frankfurters during storage with strawberry extracts. The authors observed that samples with strawberry extracts exhibited lower TBARs values after the storage period than negative control samples. Additionally, lipid-derived volatiles analysis also demonstrated that the strawberry extract inhibited the formation of certain volatile compounds and protected proteins against oxidation. Ganhão et al. [40] studied the lipid oxidation in pork patties during cooking and chilled storage by measuring TBAR values after blackberry addition. Compared to the control samples, the pork patties with blackberry extracts exhibited significantly lower TBAR values. Püssa et al. [41] experimented with the addition of plant powder extract into minced pork meat stored for eight days. They used various fruits, such as Siberian rhubarb, black currant, chokeberry, and blue honeysuckle, in 2% concentrations. Honeysuckle was by far the most effective at reducing the concentration of malondialdehyde. The addition of 2% honeysuckle addition as effective as the combination of NaCl + NaNO_2_ (1 + 1%). Anton et al. [42] also used a wide variety of plant powders incorporated into raw and cooked minced-meat pork. The results showed that tomato, rhubarb petioles, and blue honeysuckle berries exerted a pro-oxidative effect in meat mixtures and contradicted our results.

## 4. Conclusions

Our study observed the effect of blackcurrant and honeysuckle extract treatment on selected characteristics of raw-cooked meat products (frankfurters), which were vacuum-packed for 21 days. The examination of the extracts’ TAC suggested that Kamchatka honeysuckle was a more potent antioxidant than blackcurrant. TBAR results also proved this suggestion. However, both extracts were able to significantly lower the MDA concentration in the samples compared to the negative control group. This fact was observable after the 14th day of storage. The antioxidant effect of both extracts was comparable to that of synthetic vitamin C. We further observed that honeysuckle was more effective, and that its oxidation-delaying ability is concentration-dependent. No negative effects of the extract addition on the textural properties, color, or pH values were observed. During a sensory evaluation at the end of the storage period, the samples with added extracts demonstrated better scores for the color and appearance parameters. On the other hand, the evaluation demonstrates the penalization of the samples with honeysuckle extract for taste characteristics due to the bitter taste of honeysuckle. Therefore, despite its ability to retard lipid oxidation, honeysuckle extract seems to be an inappropriate option for meat products. Blackcurrant extracts seem to be more suitable for utilization in the meat industry, and more studies should be carried out to further test this option.

## Figures and Tables

**Table 1:** Ingredients used to prepare 1kg of final product.

Ingredient (g)	Con-0	Con-C	BCE-1	BCE-2	KHE-1	KHE-2
Pork meat	930	930	930	930	930	930
Water	200	200	200	200	200	200
Black pepper	2	2	2	2	2	2
Paprika (sweet)	2	2	2	2	2	2
Paprika (spicy)	2	2	2	2	2	2
Salting mixture	2	2	2	2	2	2
Nutmeg	0.5	0.5	0.5	0.5	0.5	0.5
Antioxidants						
Citric acid (g)	-	0.5	-	-	-	-
Black currant extract (mL)	-	-	3 (1.2 g)	5 (2.0 g)	-	-
Honeysuckle extract (mL)	-	-	-	-	3 (1.2 g)	5 (2.0 g)

Note: “-” in table represent absence of individual ingredient.

**Table 2:** Selected chemical characteristics of plant extracts (expressed as value ± standard deviation).

	Dry Matter (%)	TAC (% of DPPH Inhibition) ± S.D.	TPC (g GAE. kg^−1^) ± S.D.	TA (mg·L^−1^) ± S.D.
Blackcurrant	78.37	75.95 ± 2.07	16.32 ± 0.85	2588.37 ± 21.90
Honeysuckle	82.00	82.12 ± 5.17	38.67 ± 0.55	4809.43 ± 61.23

**Table 3:** Change in pH of analyzed products over time of storage.

Group	Day 1	Day 7	Day 14	Day 21
Con-0	6.28 ± 0.08 ^a^	6.29 ± 0.07 ^a^	6.28 ± 0.07 ^a^	6.26 ± 0.06 ^a^
Con-C	5.48 ± 0.07 ^b^	5.64 ± 0.03 ^b^	5.64 ± 0.03 ^b^	5.67 ± 0.04 ^b^
BCE-1	6.21 ± 0.03 ^a^	6.23 ± 0.04 ^a^	6.15 ± 0.07 ^a^	6.22 ± 0.09 ^a^
BCE-2	6.16 ± 0.05 ^a^	6.16 ± 0.09 ^a^	6.14 ± 0.09 ^a^	6.17 ± 0.08 ^a^
KHE-1	6.24 ± 0.02 ^a^	6.20 ± 0.05 ^a^	6.20 ± 0.03 ^a^	6.14 ± 0.11 ^a^
KHE-2	6.15 ± 0.06 ^a^	6.20 ± 0.05 ^a^	6.13 ± 0.12 ^a^	6.17 ± 0.14 ^a^
*p*-value	<0.001	<0.001	<0.001	<0.001

Note: Con-0 = negative control, Con-C = control with 0.5 g vit. C, BCE-1 = 3 mL blackcurrant ext. addition, BCE-2 = 5 mL blackcurrant ext. addition, KHE-1 = 3 mL honeysuckle ext. addition, KHE-2 = 5 mL honeysuckle ext. addition; results are expressed as pH value ± S.D.; a,b as upper index represent a statistically significant difference between samples in columns.

**Table 4:** Results of color measurement expressed in coordinates in color spectrum.

Group	Day 1				Day 21			
	L* (D65)	a* (D65)	b* (D65)	Chroma	L* (D65)	a* (D65)	b* (D65)	Chroma
Con-0	71.27 ± 0.51 ^a^	11.73 ± 0.35 ^a^	21.14 ± 0.36 ^a^	24.18 ± 0.20 ^a^	71.08 ± 0.05^b^	12.93 ± 0.34 ^b^	19.05 ± 0.20 ^d^	25.04 ± 0.07 ^e^
Con-C	69.90 ± 1.80 ^ab^	12.25 ± 0.36 ^a^	20.57 ± 0.60 ^ab^	23.94 ± 0.68 ^a^	73.37 ± 0.97 ^a^	13.19 ± 0.26 ^a^	21.28 ± 0.20 ^a^	22.89 ± 0.31 ^a^
BCE-1	69.52 ± 0.06 ^ab^	12.34 ± 0.30 ^a^	20.24 ± 0.30 ^ab^	23,71 ± 0.40 ^a^	69.40 ± 0.03^c^	12.95 ± 0.17 ^b^	20.68 ± 0.12 ^b^	24.40 ± 0.19 ^b^
BCE-2	69.39 ± 0.96 ^ab^	12.15 ± 0.29 ^a^	20.46 ± 0.32 ^ab^	23.80 ± 0.40 ^a^	70.56 ± 0.33 ^b^	12.95 ± 0.18 ^b^	20.25 ± 0.06 ^c^	23.37 ± 0.12 ^d^
KHE-1	69.84 ± 0.45 ^b^	12.13 ± 0.34 ^a^	20.43 ± 0.15 ^ab^	23.76 ± 0.30 ^a^	67.88 ± 0.27 ^d^	12.95 ± 0.19 ^b^	20.93 ± 0.14 ^b^	24.83 ± 0.30 ^b^
KHE-2	68.03 ± 0.43 ^b^	12.10 ± 0.26 ^a^	19.76 ± 0.45 ^b^	23.17 ± 0.42 ^a^	68.83 ± 0.23 ^c^	12.95 ± 0.20 ^b^	20.05 ± 0.30 ^c^	23.87 ± 0.07 ^c^
*p*-value	0.008	0.304	0.013	0.155	<0.001	0.039	<0.001	<0.001

Note: L* = measured lightness, a* = measured redness, b* = measured yellowness, D65 = standard illumination used for measurement; a,b,c,d as upper index represent a statistically significant difference between samples in columns.

**Table 5:** Results of microbial examination of samples.

Sample	Microorganisms	After 7th Day	After 14th Day	After 21st Day
Con-0	*Enterococcus* sp.	1.48	0.00	0.00
	*Lactobacillus* sp.	0.00	3.15	3.70
	*Bacillus* sp.	1.48	3.49	3.08
	Psychrotrophic microorganisms	2.58	0.00	1.30
Con-C	*Enterococcus* sp.	1.48	0.00	0.00
	*Lactobacillus* sp.	0.00	2.70	3.49
	*Bacillus* sp.	1.90	3.04	3.08
	Psychrotrophic microorganisms	1.48	1.48	0.00
BCE-1	*Enterococcus* sp.	0.00	0.00	0.00
	*Lactobacillus* sp.	0.00	3.29	3.48
	*Bacillus* sp.	2.04	2.30	2.95
	Psychrotrophic microorganisms	0.00	0.00	0.00
BCE-2	*Enterococcus* sp.	0.00	0.00	0.00
	*Lactobacillus* sp.	2.08	3.18	3.04
	*Bacillus* sp.	0.00	3.00	3.48
	Psychrotrophic microorganisms	0.00	0.00	2.08
KHE-1	*Enterococcus* sp.	0.00	0.00	0.00
	*Lactobacillus* sp.	1.30	2.60	3.00
	*Bacillus* sp.	0.00	2.70	3.08
	*Psychrotrophic* microorganisms	0.00	0.00	2.30
KHE-2	*Enterococcus* sp.	0.00	0.00	0.00
	*Lactobacillus* sp.	1.30	3.45	3.18
	*Bacillus* sp.	0.00	2.85	3.11
	Psychrotrophic microorganisms	0.00	0.00	2.00

Note: Con-0 = negative control, Con-C = control with 0.5 g vit. C, BCE-1 = 3 mL blackcurrant ext. addition, BCE-2 = 5 mL blackcurrant ext. addition, KHE-1 = 3 mL honeysuckle ext. addition, KHE-2 = 5 mL honeysuckle ext. addition; results are expressed as log CFU (colony forming units)·g^−1^.

**Table 6:** Effect of extract addition on production of malondialdehyde (MDA) expressed as MDA concentration in samples (mg·kg^−1^ ± standard deviation).

Group	Day 1	Day 7	Day 14	Day 21
Con-0	0.151 ± 0.006 ^a^	0.167 ± 0.003 ^a^	0.202 ± 0.006 ^a^	0.373 ± 0.006 ^a^
Con-C	0.133 ± 0.002 ^a^	0.149 ± 0.006 ^a^	0.167 ± 0.011 ^b^	0.208 ± 0.009 ^bc^
BCE-1	0.144 ± 0.005 ^a^	0.147 ± 0.007 ^a^	0.174 ± 0.005 ^b^	0.226 ± 0.005 ^b^
BCE-2	0.141 ± 0.009 ^a^	0.142 ± 0.011 ^a^	0.161 ± 0.005 ^b^	0.199 ± 0.004 ^bc^
KHE-1	0.142 ± 0.007 ^a^	0.143 ± 0.015 ^a^	0.170 ± 0.013 ^b^	0.220 ± 0.003 ^bc^
KHE-2	0.140 ± 0.004 ^a^	0.147 ± 0.002 ^a^	0.166 ± 0.007 ^b^	0.195 ± 0.016 ^c^
*p*-value	0.127	0.140	0.004	<0.001

Note: a,b,c as upper index represent statistically significant differences (α = 0.05) between samples in the column after ANOVA analysis using Duncan test. Con-0 = negative control, Con-C = control with 0.5 g vit. C, BCE-1 = 3 mL blackcurrant ext. addition, BCE-2 = 5 mL blackcurrant ext. addition, KHE-1 = 3 mL honeysuckle ext. addition, KHE-2 = 5 mL honeysuckle ext. addition.
